# Alteration of Sensibility in Articular Rheumatism, and the Electro-Therapeutical Treatment of the Disease

**Published:** 1876-03

**Authors:** 


### Alterations of Sensibility in Articular Rheumatism, and the Electro-Therapeutical Treatment of the Disease.

Dr. Drosdoff has called attention to a very remarkable reduction of sensibility to the action of the electric current that he observed in a case of acute articular rheumatism, though both the pain in movement and when pressure was made were very considerable, from which he concludes that there are separate channels for the conduction of different kinds of stimuli, and that the perception of various kins of sensation should be more carefully investigated in other cases. For faradisation he recommends the use of Dubois Reymond's apparatus with Grove's elements. The feeling of space he tests by means of Weber's circle method, and the perception of pressure by Wright's plan. The sensation of temperature may be tested by means of metal plates dipped in water at different temperatures. In the case he records, the application of very severe shocks of electricity was not perceived, though lively pain was experienced on the slightest pressure. It was curious to observe that the special diminution of perceptivity was strictly limited to the parts affected with the disease—just beyond these parts the sensibility was as acute as elsewhere. The reduction of sensibility for electric excitation begins, he thinks, earlier, and remains longer than the pains in the joints, and there is no prospect of permanent recovery as long as the electro-cutaneous excitability has not returned to its normal amount. Dr. Drosdoff observed, that coincidently with the diminution of the electro-cutaneous sensibility, the faculty of perceiving pressure was exalted. The tacitle sensibility is augmented in acuteness, but also in many cases undergoes abnormal variations. The increased thermic and tactile sensibility is diminished after from five to ten minutes' persistent faradisation. The cutaneous temperature over the affected joints is constantly two or three degrees Centigrade higher than in the parts in the vicinity. This exaltation of temperature, as a rule, precedes rheumatic pains, and lasts after they have departed. It also is reduced by faradisation applied for a few minutes, and the reduction lasts for several hours. The application of faradisation does protect the patient from a relapse, but it materially shortens the duration of an attack and renders it much milder. Drosdoff consequently strongly recommends that it should be employed as one of the means in the treatment of acute articular rheumatism.—(*Botkin's Klinick zu St. Petersburg und Rundschau*, Oct. and Nov., 1875.

